# Tris(1,10-phenanthroline-κ^2^ *N*,*N*′)zinc(II) bis­(4-bromo­benzoate) 6.5-hydrate

**DOI:** 10.1107/S1600536810010561

**Published:** 2010-03-31

**Authors:** Su-Fang Ye, Bi-Song Zhang

**Affiliations:** a College of Material Science and Chemical Engineering, Jinhua College of Profession and Technology, Jinhua, Zhejiang 321017, People’s Republic of China

## Abstract

In the title compound, [Zn(C_12_H_8_N_2_)_3_](C_7_H_4_BrO_2_)_2_·6.5H_2_O, the Zn^II^ atom is coordinated by six N atoms from three 1,10-phenanthroline (phen) mol­ecules in a distorted octa­hedral geometry. The chelating phen ligands exhibit nearly perfect coplanarity (r.m.s. deviations of 0.048, 0.039 and 0.061 Å). The mean inter­planar distances of 3.51 (2) and 3.54 (4) Å between adjacent phen ligands indicate π–π stacking inter­actions, which connect the complex cations into chains along [101]. The 4-bromo­benzoate anions and the uncoordinated water mol­ecules, parts of which are not fully occupied, are linked by O—H⋯O hydrogen bonds. Two carboxyl­ate O atoms and one Br atom in the 4-bromo­benzoate anions are each disordered over two sites with occupancy factors of 0.60 and 0.40.

## Related literature

For other zinc(II) complexes with 1,10-phenanthroline ligands, see: Aghabozorg *et al.* (2005 ▶); Chen *et al.* (2006 ▶); Liu *et al.* (1998 ▶); Wei, Yuan *et al.* (2004 ▶); Wei, Zheng *et al.* (2002 ▶).
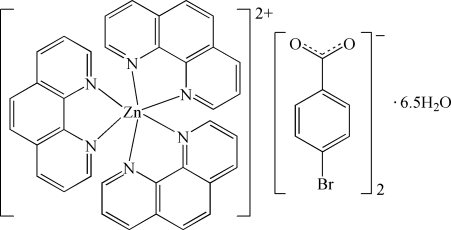


## Experimental

### 

#### Crystal data

[Zn(C_12_H_8_N_2_)_3_](C_7_H_4_BrO_2_)_2_·6.5H_2_O
*M* *_r_* = 1123.11
Triclinic, 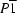

*a* = 13.098 (3) Å
*b* = 14.240 (3) Å
*c* = 16.281 (3) Å
α = 108.68 (3)°
β = 107.13 (3)°
γ = 105.11 (3)°
*V* = 2528.9 (15) Å^3^
*Z* = 2
Mo *K*α radiation
μ = 2.13 mm^−1^
*T* = 290 K
0.28 × 0.20 × 0.19 mm

#### Data collection

Rigaku R-AXIS RAPID diffractometer
Absorption correction: multi-scan (*ABSCOR*; Higashi, 1995 ▶) *T*_min_ = 0.603, *T*_max_ = 0.675
19484 measured reflections
8780 independent reflections
5375 reflections with *I* > 2σ(*I*)
*R*_int_ = 0.039

#### Refinement

*R*[*F* ^2^ > 2σ(*F* ^2^)] = 0.065
*wR*(*F* ^2^) = 0.250
*S* = 1.15
8780 reflections
667 parameters
9 restraints
H-atom parameters constrained
Δρ_max_ = 0.80 e Å^−3^
Δρ_min_ = −0.95 e Å^−3^

### 

Data collection: *PROCESS-AUTO* (Rigaku, 1998 ▶); cell refinement: *PROCESS-AUTO*; data reduction: *PROCESS-AUTO*; program(s) used to solve structure: *SHELXS97* (Sheldrick, 2008 ▶); program(s) used to refine structure: *SHELXL97* (Sheldrick, 2008 ▶); molecular graphics: *SHELXTL* (Sheldrick, 2008 ▶); software used to prepare material for publication: *SHELXTL*.

## Supplementary Material

Crystal structure: contains datablocks I, global. DOI: 10.1107/S1600536810010561/hy2291sup1.cif

Structure factors: contains datablocks I. DOI: 10.1107/S1600536810010561/hy2291Isup2.hkl

Additional supplementary materials:  crystallographic information; 3D view; checkCIF report

## Figures and Tables

**Table 1:** Hydrogen-bond geometry (Å, °)

*D*—H⋯*A*	*D*—H	H⋯*A*	*D*⋯*A*	*D*—H⋯*A*
O5—H5*A*⋯O2	0.82	1.90	2.700 (11)	164
O5—H5*B*⋯O6^i^	0.82	2.23	2.676 (11)	114
O6—H6*A*⋯O3	0.82	1.97	2.763 (2)	162
O6—H6*A*⋯O3′	0.82	2.03	2.767 (5)	150
O6—H6*B*⋯O8^ii^	0.82	2.20	2.788 (5)	129
O7—H7*A*⋯O5^i^	0.82	1.97	2.787 (10)	176
O7—H7*B*⋯O4	0.82	1.89	2.687 (11)	163
O8—H8*A*⋯O3	0.82	2.04	2.803 (2)	155
O8—H8*A*⋯O3′	0.82	1.99	2.789 (2)	166
O8—H8*B*⋯O1^iii^	0.82	2.19	2.862 (5)	139
O8—H8*B*⋯O1′^iii^	0.82	1.96	2.685 (6)	146
O10—H10*A*⋯O11^iv^	0.82	2.24	2.806 (2)	126
O10—H10*B*⋯O2	0.82	2.11	2.739 (2)	134
O11—H11*A*⋯O5	0.82	2.27	2.826 (5)	126
O11—H11*B*⋯O10^iv^	0.82	2.33	2.806 (2)	117
O12—H12*A*⋯O13^i^	0.82	2.50	2.981 (5)	118
O12—H12*B*⋯O4	0.82	2.29	2.786 (2)	119
O13—H13*A*⋯O12^v^	0.82	2.19	2.796 (5)	131
O13—H13*B*⋯O7^i^	0.82	1.95	2.746 (2)	165

Symmetry codes: (i) 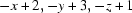; (ii) 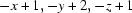; (iii) 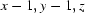; (iv) 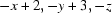; (v) 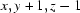.

**Fig. 1.**
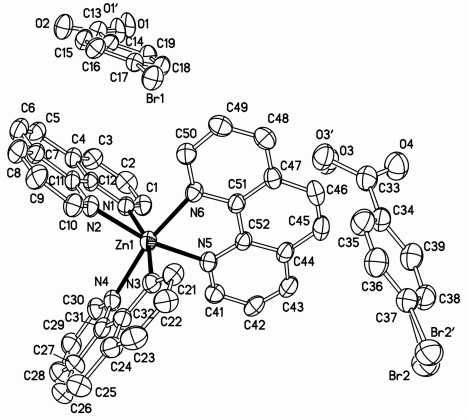
The molecular structure of the title compound. Displacement ellipsoids are drawn at the 50% probability level. H atoms and uncoordinated water molecules are omitted for clarity.

**Fig. 2.**
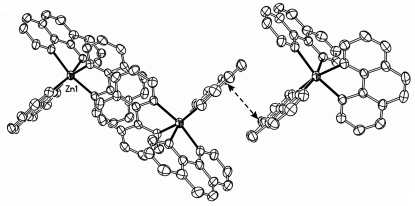
The π–π stacking interactions (dashed double arrows), with the mean interplanar distances of 3.51 (2) and 3.54 (4)Å.

## supplementary crystallographic information

### Comment

Zinc ions with 1,10-phenanthroline (phen) ligands can form tris(phen)zinc(II) (Aghabozorg *et al.*, 2005; Chen *et al.*, 2006; Liu *et al.*, 1998; Wei, Yuan *et al.*, 2004; Wei, Zheng *et al.*, 2002). In this paper, we report the synthesis and structure of a tris(phen)zinc(II) complex.

The title compound consists of [Zn(phen)_3_]^2+^ complex cations, 4-bromobenzoate anions and uncoordinated water molecules (Fig. 1). In the cation, the Zn^II^ atom is coordinated by six N atoms from three phen molecules to complete a distorted ZnN_6_ octahedral geometry. The Zn—N bond lengths are in the range of 2.126 (6)–2.199 (6)Å. The chelating phen ligands exhibit nearly perfect coplanarity. The mean interplanar distances of 3.51 (2) and 3.54 (4)Å between adjacent phen ligands indicate π–π stacking interactions (Fig. 2). The complex cations are connected to each other via π–π stacking interactions into a chain along [1 0 1]. The 4-bromobenzoate anions and the uncoordinated water molecules are linked by O—H···O hydrogen bonds (Table 1).

### Experimental

ZnSO_4_.7H_2_O (0.237 g, 0.826 mmol) was dissolved in appropriate amount of water and then 1M Na_2_CO_3_ solution was added. ZnCO_3_ was obtained by filtration and washed with distilled water for 5 times. The freshly prepared ZnCO_3_, phen.H_2_O (0.0496 g, 0.25 mmol) and 4-bromobenzoic acid (0.050 g, 0.25 mmol) were mixed in CH_3_OH/H_2_O (15 ml, v/v = 1:2) and stirred for 2 h. The resulting cream suspension was heated in a 23 ml Teflon-lined stainless steel autoclave at 433 K for 97 h. After the autoclave was cooled to room temperature in 43 h, the solid was filtered off. The resulting filtrate was allowed to stand at room temperature, and slow evaporation for 6 months afforded brown block single crystals.

### Refinement

C-bound H atoms were placed in calculated positions and refined using a riding model, with C—H = 0.93 Å and *U*_iso_(H) = 1.2*U*_eq_(C). H atoms of the water molecules were located in a difference Fourier map and refined as riding, with O—H = 0.82 Å and *U*_iso_(H) = 1.5*U*_eq_(O). Two carboxylate O atoms (O1 and O3) and one Br atom (Br2) are each disordered over two sites with occupancy factors of 0.60 and 0.40. Four water molecules (O10, O11, O12 and O13) are half-occupied and the two water molecules (O9 and O14) are quarter-occupied.

### Crystal data

**Table:**

[Zn(C_12_H_8_N_2_)_3_](C_7_H_4_BrO_2_)_2_·6.5H_2_O	*Z* = 2
*M**_r_* = 1123.11	*F*(000) = 1142
Triclinic, *P*1	*D*_x_ = 1.475 Mg m^−^^3^
Hall symbol: -P 1	Mo *K*α radiation, λ = 0.71073 Å
*a* = 13.098 (3) Å	Cell parameters from 13525 reflections
*b* = 14.240 (3) Å	θ = 3.1–25.0°
*c* = 16.281 (3) Å	µ = 2.13 mm^−^^1^
α = 108.68 (3)°	*T* = 290 K
β = 107.13 (3)°	Block, yellow
γ = 105.11 (3)°	0.28 × 0.20 × 0.19 mm
*V* = 2528.9 (15) Å^3^	

### Data collection

**Table:**

Rigaku R-AXIS RAPID diffractometer	8780 independent reflections
Radiation source: fine-focus sealed tube	5375 reflections with *I* > 2σ(*I*)
graphite	*R*_int_ = 0.039
ω scans	θ_max_ = 25.0°, θ_min_ = 3.1°
Absorption correction: multi-scan (*ABSCOR*; Higashi, 1995)	*h* = −15→15
*T*_min_ = 0.603, *T*_max_ = 0.675	*k* = −16→16
19484 measured reflections	*l* = −19→17

### Refinement

**Table:**

Refinement on *F*^2^	Primary atom site location: structure-invariant direct methods
Least-squares matrix: full	Secondary atom site location: difference Fourier map
*R*[*F*^2^ > 2σ(*F*^2^)] = 0.065	Hydrogen site location: inferred from neighbouring sites
*wR*(*F*^2^) = 0.250	H-atom parameters constrained
*S* = 1.15	*w* = 1/[σ^2^(*F*_o_^2^) + (0.1215*P*)^2^ + 3.2158*P*] where *P* = (*F*_o_^2^ + 2*F*_c_^2^)/3
8780 reflections	(Δ/σ)_max_ < 0.001
667 parameters	Δρ_max_ = 0.80 e Å^−^^3^
9 restraints	Δρ_min_ = −0.95 e Å^−^^3^

### Fractional atomic coordinates and isotropic or equivalent isotropic displacement parameters (Å^2^)

**Table:**

	*x*	*y*	*z*	*U*_iso_*/*U*_eq_	Occ. (<1)
Zn1	0.67519 (7)	0.86835 (7)	0.19869 (5)	0.0560 (3)	
N1	0.7784 (5)	0.9984 (5)	0.1831 (4)	0.0580 (14)	
N2	0.5521 (5)	0.9405 (5)	0.1550 (4)	0.0587 (14)	
N3	0.5319 (5)	0.7306 (5)	0.1766 (4)	0.0585 (14)	
N4	0.6269 (5)	0.7512 (5)	0.0531 (4)	0.0600 (14)	
N5	0.8131 (5)	0.8264 (5)	0.2669 (4)	0.0576 (14)	
N6	0.7160 (5)	0.9524 (4)	0.3480 (4)	0.0538 (13)	
Br1	0.83384 (7)	1.27529 (7)	0.40614 (7)	0.0820 (3)	
Br2	0.3576 (11)	0.3728 (8)	0.5774 (7)	0.0897 (12)	0.60
O1	1.2712 (11)	1.617 (5)	0.377 (3)	0.087 (5)	0.60
Br2'	0.3469 (17)	0.3886 (13)	0.5953 (11)	0.0897 (12)	0.40
O1'	1.2774 (16)	1.629 (8)	0.362 (6)	0.087 (5)	0.40
O2	1.1233 (5)	1.6134 (5)	0.2571 (5)	0.0925 (18)	
O3	0.6296 (19)	0.892 (3)	0.631 (3)	0.087 (6)	0.60
O3'	0.597 (3)	0.879 (5)	0.636 (5)	0.087 (6)	0.40
O4	0.7686 (6)	0.8797 (5)	0.7350 (5)	0.0960 (19)	
O5	1.1945 (5)	1.7751 (5)	0.2083 (4)	0.0920 (18)	
H5A	1.1640	1.7193	0.2118	0.138*	
H5B	1.2580	1.7764	0.2098	0.138*	
O6	0.5911 (5)	1.0804 (5)	0.6688 (4)	0.0845 (16)	
H6A	0.6177	1.0337	0.6620	0.127*	
H6B	0.5542	1.0700	0.6141	0.127*	
O7	0.9162 (5)	1.0824 (5)	0.7940 (4)	0.0891 (17)	
H7A	0.8820	1.1226	0.7903	0.134*	
H7B	0.8655	1.0264	0.7829	0.134*	
O8	0.4664 (5)	0.8127 (5)	0.4431 (4)	0.0823 (15)	
H8A	0.4977	0.8216	0.4983	0.124*	
H8B	0.4086	0.7618	0.3992	0.124*	
O9	0.051 (3)	0.206 (3)	−0.012 (2)	0.107 (9)	0.25
H9B	0.0769	0.2258	0.0461	0.160*	0.25
H9A	0.0745	0.2430	−0.0369	0.160*	0.25
O10	0.9611 (10)	1.5048 (9)	0.0723 (7)	0.087 (3)	0.50
H10A	0.8976	1.4562	0.0520	0.130*	0.50
H10B	1.0168	1.5063	0.1131	0.130*	0.50
O11	1.1443 (9)	1.7210 (8)	0.0139 (7)	0.075 (3)	0.50
H11A	1.1859	1.7720	0.0662	0.112*	0.50
H11B	1.0849	1.6786	0.0098	0.112*	0.50
O12	0.8335 (7)	0.9090 (9)	0.9234 (6)	0.065 (3)	0.50
H12A	0.8195	0.9575	0.9119	0.097*	0.50
H12B	0.8037	0.8562	0.8719	0.097*	0.50
O13	1.0171 (17)	1.8620 (16)	0.0149 (10)	0.121 (6)	0.50
H13A	1.0012	1.9048	−0.0050	0.182*	0.50
H13B	1.0440	1.8717	0.0706	0.182*	0.50
O14	0.097 (2)	0.339 (3)	−0.031 (2)	0.136 (12)	0.25
H14A	0.0700	0.3688	0.0043	0.204*	0.25
H14B	0.0535	0.3342	−0.0816	0.204*	0.25
C1	0.8918 (6)	1.0293 (6)	0.1990 (5)	0.0642 (18)	
H1	0.9320	0.9897	0.2185	0.077*	
C2	0.9512 (7)	1.1167 (7)	0.1878 (6)	0.076 (2)	
H2	1.0291	1.1348	0.1991	0.091*	
C3	0.8929 (7)	1.1769 (7)	0.1595 (6)	0.075 (2)	
H3	0.9315	1.2364	0.1522	0.090*	
C4	0.7765 (6)	1.1478 (6)	0.1424 (5)	0.0627 (18)	
C5	0.7082 (8)	1.2060 (7)	0.1135 (6)	0.077 (2)	
H5	0.7413	1.2625	0.1003	0.093*	
C6	0.6007 (8)	1.1811 (7)	0.1055 (6)	0.077 (2)	
H6	0.5615	1.2233	0.0906	0.092*	
C7	0.5417 (7)	1.0906 (6)	0.1190 (5)	0.0653 (19)	
C8	0.4278 (7)	1.0608 (7)	0.1102 (6)	0.075 (2)	
H8	0.3847	1.1000	0.0947	0.090*	
C9	0.3798 (7)	0.9734 (7)	0.1246 (6)	0.078 (2)	
H9	0.3044	0.9540	0.1206	0.094*	
C10	0.4431 (6)	0.9139 (6)	0.1450 (5)	0.0664 (19)	
H10	0.4079	0.8529	0.1519	0.080*	
C11	0.6028 (6)	1.0283 (6)	0.1431 (4)	0.0541 (16)	
C12	0.7218 (6)	1.0589 (6)	0.1556 (4)	0.0538 (16)	
C13	1.1649 (7)	1.5866 (7)	0.3201 (6)	0.070 (2)	
C14	1.0849 (6)	1.5085 (6)	0.3391 (5)	0.0595 (17)	
C15	0.9672 (7)	1.4864 (6)	0.3041 (6)	0.071 (2)	
H15	0.9374	1.5167	0.2648	0.085*	
C16	0.8927 (7)	1.4199 (6)	0.3264 (6)	0.070 (2)	
H16	0.8144	1.4085	0.3052	0.084*	
C17	0.9367 (7)	1.3716 (6)	0.3801 (5)	0.0625 (18)	
C18	1.0531 (7)	1.3907 (6)	0.4151 (6)	0.071 (2)	
H18	1.0818	1.3575	0.4518	0.085*	
C19	1.1268 (6)	1.4602 (7)	0.3946 (5)	0.070 (2)	
H19	1.2057	1.4744	0.4188	0.084*	
C21	0.4868 (6)	0.7190 (6)	0.2371 (5)	0.0628 (18)	
H21	0.5278	0.7675	0.3010	0.075*	
C22	0.3804 (7)	0.6374 (7)	0.2098 (6)	0.073 (2)	
H22	0.3519	0.6318	0.2548	0.088*	
C23	0.3192 (8)	0.5664 (7)	0.1168 (7)	0.082 (2)	
H23	0.2471	0.5128	0.0969	0.098*	
C24	0.3663 (7)	0.5747 (6)	0.0502 (6)	0.071 (2)	
C25	0.3092 (8)	0.5004 (7)	−0.0495 (7)	0.088 (3)	
H25	0.2362	0.4464	−0.0727	0.105*	
C26	0.3592 (9)	0.5079 (7)	−0.1090 (6)	0.087 (3)	
H26	0.3213	0.4586	−0.1727	0.104*	
C27	0.4715 (7)	0.5915 (6)	−0.0753 (5)	0.070 (2)	
C28	0.5267 (9)	0.6002 (8)	−0.1358 (6)	0.084 (2)	
H28	0.4932	0.5503	−0.1993	0.100*	
C29	0.6320 (9)	0.6848 (8)	−0.0987 (6)	0.082 (3)	
H29	0.6718	0.6915	−0.1367	0.098*	
C30	0.6785 (7)	0.7599 (7)	−0.0047 (5)	0.069 (2)	
H30	0.7482	0.8183	0.0187	0.083*	
C31	0.5244 (7)	0.6672 (6)	0.0182 (5)	0.0612 (18)	
C32	0.4730 (6)	0.6576 (6)	0.0832 (5)	0.0611 (17)	
C33	0.6651 (8)	0.8397 (7)	0.6753 (6)	0.079 (2)	
C34	0.5895 (7)	0.7272 (6)	0.6533 (5)	0.0666 (19)	
C35	0.4714 (7)	0.6959 (7)	0.6280 (6)	0.078 (2)	
H35	0.4376	0.7450	0.6234	0.094*	
C36	0.4020 (7)	0.5930 (7)	0.6092 (6)	0.078 (2)	
H36	0.3233	0.5745	0.5950	0.093*	
C37	0.4492 (7)	0.5199 (6)	0.6117 (5)	0.070 (2)	
C38	0.5672 (8)	0.5469 (7)	0.6357 (6)	0.073 (2)	
H38	0.5994	0.4961	0.6371	0.088*	
C39	0.6360 (7)	0.6512 (7)	0.6575 (6)	0.076 (2)	
H39	0.7155	0.6706	0.6755	0.091*	
C41	0.8658 (6)	0.7683 (6)	0.2270 (5)	0.0643 (18)	
H41	0.8496	0.7488	0.1629	0.077*	
C42	0.9437 (7)	0.7362 (6)	0.2782 (6)	0.070 (2)	
H42	0.9800	0.6971	0.2489	0.084*	
C43	0.9662 (7)	0.7622 (6)	0.3703 (6)	0.071 (2)	
H43	1.0158	0.7382	0.4039	0.086*	
C44	0.9159 (6)	0.8252 (6)	0.4169 (5)	0.0628 (18)	
C45	0.9371 (7)	0.8596 (7)	0.5155 (6)	0.076 (2)	
H45	0.9854	0.8374	0.5525	0.091*	
C46	0.8895 (7)	0.9221 (7)	0.5550 (6)	0.075 (2)	
H46	0.9057	0.9429	0.6193	0.091*	
C47	0.8146 (6)	0.9586 (6)	0.5024 (5)	0.0597 (17)	
C48	0.7702 (7)	1.0302 (7)	0.5428 (6)	0.071 (2)	
H48	0.7860	1.0556	0.6072	0.085*	
C49	0.7014 (7)	1.0631 (7)	0.4847 (6)	0.072 (2)	
H49	0.6727	1.1130	0.5106	0.087*	
C50	0.6757 (6)	1.0222 (6)	0.3889 (5)	0.0633 (18)	
H50	0.6281	1.0443	0.3511	0.076*	
C51	0.7871 (6)	0.9222 (5)	0.4041 (5)	0.0561 (16)	
C52	0.8387 (6)	0.8548 (5)	0.3610 (5)	0.0523 (15)	

### Atomic displacement parameters (Å^2^)

**Table:**

	*U*^11^	*U*^22^	*U*^33^	*U*^12^	*U*^13^	*U*^23^
Zn1	0.0597 (5)	0.0667 (5)	0.0523 (5)	0.0308 (4)	0.0242 (4)	0.0325 (4)
N1	0.066 (4)	0.066 (4)	0.052 (3)	0.035 (3)	0.025 (3)	0.029 (3)
N2	0.065 (4)	0.073 (4)	0.053 (3)	0.034 (3)	0.026 (3)	0.038 (3)
N3	0.068 (4)	0.064 (4)	0.053 (3)	0.032 (3)	0.029 (3)	0.029 (3)
N4	0.066 (4)	0.076 (4)	0.052 (3)	0.039 (3)	0.027 (3)	0.033 (3)
N5	0.059 (3)	0.065 (4)	0.057 (3)	0.026 (3)	0.026 (3)	0.033 (3)
N6	0.059 (3)	0.063 (3)	0.058 (3)	0.030 (3)	0.032 (3)	0.035 (3)
Br1	0.0786 (6)	0.0848 (6)	0.0949 (6)	0.0271 (5)	0.0398 (5)	0.0536 (5)
Br2	0.110 (2)	0.070 (3)	0.081 (3)	0.0233 (16)	0.0395 (18)	0.032 (2)
O1	0.067 (4)	0.106 (13)	0.103 (16)	0.018 (5)	0.035 (5)	0.075 (7)
Br2'	0.110 (2)	0.070 (3)	0.081 (3)	0.0233 (16)	0.0395 (18)	0.032 (2)
O1'	0.067 (4)	0.106 (13)	0.103 (16)	0.018 (5)	0.035 (5)	0.075 (7)
O2	0.079 (4)	0.106 (5)	0.112 (5)	0.034 (3)	0.039 (4)	0.073 (4)
O3	0.093 (14)	0.070 (10)	0.085 (6)	0.015 (14)	0.020 (12)	0.048 (5)
O3'	0.093 (14)	0.070 (10)	0.085 (6)	0.015 (14)	0.020 (12)	0.048 (5)
O4	0.082 (4)	0.096 (5)	0.093 (4)	0.022 (4)	0.020 (4)	0.047 (4)
O5	0.095 (4)	0.093 (4)	0.109 (5)	0.044 (4)	0.042 (4)	0.064 (4)
O6	0.084 (4)	0.096 (4)	0.087 (4)	0.042 (3)	0.039 (3)	0.046 (3)
O7	0.079 (4)	0.102 (5)	0.104 (4)	0.042 (3)	0.041 (3)	0.058 (4)
O8	0.088 (4)	0.082 (4)	0.076 (4)	0.035 (3)	0.027 (3)	0.038 (3)
O9	0.11 (2)	0.13 (3)	0.10 (2)	0.07 (2)	0.035 (18)	0.07 (2)
O10	0.073 (7)	0.078 (7)	0.063 (6)	0.005 (6)	0.006 (5)	0.017 (6)
O11	0.087 (7)	0.071 (7)	0.060 (6)	0.012 (6)	0.032 (5)	0.033 (5)
O12	0.056 (5)	0.105 (8)	0.055 (5)	0.037 (5)	0.030 (5)	0.049 (6)
O13	0.169 (17)	0.150 (16)	0.066 (8)	0.092 (14)	0.039 (10)	0.058 (11)
O14	0.063 (16)	0.13 (3)	0.13 (2)	−0.008 (16)	0.029 (16)	0.00 (2)
C1	0.059 (4)	0.084 (5)	0.065 (4)	0.039 (4)	0.032 (4)	0.035 (4)
C2	0.062 (5)	0.089 (6)	0.081 (5)	0.021 (4)	0.042 (4)	0.038 (5)
C3	0.081 (6)	0.074 (5)	0.089 (6)	0.032 (4)	0.047 (5)	0.047 (5)
C4	0.069 (5)	0.074 (5)	0.059 (4)	0.035 (4)	0.028 (4)	0.038 (4)
C5	0.099 (7)	0.081 (6)	0.082 (5)	0.047 (5)	0.045 (5)	0.054 (5)
C6	0.092 (6)	0.083 (6)	0.081 (5)	0.048 (5)	0.037 (5)	0.052 (5)
C7	0.077 (5)	0.077 (5)	0.060 (4)	0.048 (4)	0.030 (4)	0.035 (4)
C8	0.063 (5)	0.087 (6)	0.086 (6)	0.043 (4)	0.026 (4)	0.044 (5)
C9	0.062 (5)	0.101 (7)	0.094 (6)	0.057 (5)	0.037 (4)	0.043 (5)
C10	0.058 (4)	0.078 (5)	0.075 (5)	0.034 (4)	0.030 (4)	0.039 (4)
C11	0.061 (4)	0.071 (4)	0.045 (3)	0.038 (4)	0.022 (3)	0.031 (3)
C12	0.064 (4)	0.065 (4)	0.044 (3)	0.033 (3)	0.023 (3)	0.030 (3)
C13	0.066 (5)	0.074 (5)	0.080 (5)	0.027 (4)	0.035 (4)	0.041 (5)
C14	0.059 (4)	0.058 (4)	0.064 (4)	0.026 (3)	0.024 (3)	0.028 (4)
C15	0.065 (5)	0.072 (5)	0.079 (5)	0.030 (4)	0.020 (4)	0.041 (4)
C16	0.062 (5)	0.071 (5)	0.089 (5)	0.030 (4)	0.034 (4)	0.043 (5)
C17	0.069 (5)	0.060 (4)	0.058 (4)	0.026 (4)	0.022 (4)	0.028 (4)
C18	0.071 (5)	0.072 (5)	0.076 (5)	0.032 (4)	0.025 (4)	0.041 (4)
C19	0.051 (4)	0.085 (5)	0.072 (5)	0.022 (4)	0.022 (4)	0.039 (4)
C21	0.070 (5)	0.070 (5)	0.069 (4)	0.034 (4)	0.040 (4)	0.037 (4)
C22	0.082 (6)	0.074 (5)	0.078 (5)	0.031 (4)	0.046 (5)	0.037 (5)
C23	0.076 (6)	0.071 (5)	0.104 (7)	0.025 (4)	0.045 (5)	0.039 (5)
C24	0.069 (5)	0.062 (5)	0.075 (5)	0.027 (4)	0.025 (4)	0.025 (4)
C25	0.087 (6)	0.071 (6)	0.080 (6)	0.021 (5)	0.022 (5)	0.021 (5)
C26	0.103 (7)	0.071 (6)	0.052 (4)	0.034 (5)	0.007 (5)	0.009 (4)
C27	0.081 (5)	0.068 (5)	0.048 (4)	0.030 (4)	0.018 (4)	0.016 (4)
C28	0.108 (7)	0.086 (6)	0.056 (5)	0.051 (6)	0.028 (5)	0.025 (5)
C29	0.115 (8)	0.102 (7)	0.059 (5)	0.063 (6)	0.050 (5)	0.041 (5)
C30	0.077 (5)	0.089 (6)	0.057 (4)	0.040 (4)	0.033 (4)	0.038 (4)
C31	0.069 (5)	0.073 (5)	0.062 (4)	0.044 (4)	0.029 (4)	0.038 (4)
C32	0.065 (4)	0.067 (5)	0.060 (4)	0.033 (4)	0.025 (4)	0.033 (4)
C33	0.093 (6)	0.078 (6)	0.070 (5)	0.031 (5)	0.032 (5)	0.040 (5)
C34	0.078 (5)	0.072 (5)	0.061 (4)	0.036 (4)	0.028 (4)	0.037 (4)
C35	0.075 (5)	0.080 (6)	0.086 (6)	0.038 (5)	0.028 (5)	0.041 (5)
C36	0.064 (5)	0.096 (6)	0.083 (5)	0.037 (5)	0.030 (4)	0.047 (5)
C37	0.086 (6)	0.065 (5)	0.060 (4)	0.027 (4)	0.029 (4)	0.032 (4)
C38	0.088 (6)	0.078 (6)	0.071 (5)	0.040 (5)	0.037 (4)	0.042 (4)
C39	0.072 (5)	0.096 (6)	0.082 (5)	0.043 (5)	0.039 (4)	0.050 (5)
C41	0.068 (5)	0.068 (5)	0.065 (4)	0.032 (4)	0.034 (4)	0.030 (4)
C42	0.065 (5)	0.069 (5)	0.099 (6)	0.040 (4)	0.039 (4)	0.046 (5)
C43	0.067 (5)	0.066 (5)	0.089 (6)	0.031 (4)	0.024 (4)	0.045 (5)
C44	0.062 (4)	0.059 (4)	0.073 (5)	0.022 (4)	0.023 (4)	0.040 (4)
C45	0.066 (5)	0.087 (6)	0.073 (5)	0.027 (4)	0.014 (4)	0.050 (5)
C46	0.069 (5)	0.096 (6)	0.054 (4)	0.024 (5)	0.017 (4)	0.038 (4)
C47	0.055 (4)	0.069 (5)	0.054 (4)	0.017 (3)	0.020 (3)	0.032 (4)
C48	0.072 (5)	0.081 (5)	0.061 (4)	0.028 (4)	0.035 (4)	0.026 (4)
C49	0.076 (5)	0.076 (5)	0.068 (5)	0.030 (4)	0.041 (4)	0.023 (4)
C50	0.062 (4)	0.076 (5)	0.064 (4)	0.035 (4)	0.032 (4)	0.033 (4)
C51	0.059 (4)	0.055 (4)	0.057 (4)	0.022 (3)	0.022 (3)	0.031 (3)
C52	0.054 (4)	0.050 (4)	0.058 (4)	0.019 (3)	0.021 (3)	0.031 (3)

### Geometric parameters (Å, °)

**Table:**

Zn1—N1	2.126 (6)	C9—C10	1.378 (10)
Zn1—N5	2.159 (6)	C9—H9	0.9300
Zn1—N6	2.160 (5)	C10—H10	0.9300
Zn1—N3	2.175 (6)	C11—C12	1.436 (9)
Zn1—N2	2.195 (5)	C13—C14	1.495 (10)
Zn1—N4	2.199 (6)	C14—C19	1.376 (10)
N1—C1	1.352 (9)	C14—C15	1.384 (10)
N1—C12	1.370 (8)	C15—C16	1.389 (10)
N2—C10	1.323 (9)	C15—H15	0.9300
N2—C11	1.356 (8)	C16—C17	1.364 (10)
N3—C21	1.321 (8)	C16—H16	0.9300
N3—C32	1.365 (9)	C17—C18	1.377 (10)
N4—C30	1.328 (9)	C18—C19	1.388 (10)
N4—C31	1.354 (9)	C18—H18	0.9300
N5—C41	1.342 (9)	C19—H19	0.9300
N5—C52	1.361 (8)	C21—C22	1.396 (10)
N6—C50	1.330 (9)	C21—H21	0.9300
N6—C51	1.358 (8)	C22—C23	1.356 (12)
Br1—C17	1.903 (7)	C22—H22	0.9300
Br2—C37	1.920 (13)	C23—C24	1.417 (11)
O1—C13	1.286 (15)	C23—H23	0.9300
Br2'—C37	1.878 (19)	C24—C32	1.388 (11)
O1'—C13	1.297 (19)	C24—C25	1.445 (11)
O2—C13	1.235 (9)	C25—C26	1.335 (12)
O3—C33	1.279 (15)	C25—H25	0.9300
O3'—C33	1.293 (19)	C26—C27	1.441 (12)
O4—C33	1.255 (10)	C26—H26	0.9300
O5—H5A	0.8200	C27—C31	1.378 (10)
O5—H5B	0.8200	C27—C28	1.399 (12)
O6—H6A	0.8200	C28—C29	1.377 (13)
O6—H6B	0.8202	C28—H28	0.9300
O7—H7A	0.8194	C29—C30	1.386 (11)
O7—H7B	0.8200	C29—H29	0.9300
O8—H8A	0.8202	C30—H30	0.9300
O8—H8B	0.8200	C31—C32	1.434 (10)
O9—O13^i^	1.11 (3)	C33—C34	1.507 (11)
O9—H9B	0.8199	C34—C35	1.382 (11)
O9—H9A	0.81000	C34—C39	1.385 (10)
O10—H10A	0.8200	C35—C36	1.386 (12)
O10—H10B	0.8198	C35—H35	0.9300
O11—H11A	0.8200	C36—C37	1.347 (11)
O11—H11B	0.8201	C36—H36	0.9300
O12—H12A	0.8200	C37—C38	1.389 (11)
O12—H12B	0.8200	C38—C39	1.386 (11)
O13—O9^i^	1.11 (3)	C38—H38	0.9300
O13—H13A	0.8200	C39—H39	0.9300
O13—H13B	0.8201	C41—C42	1.389 (10)
O14—H9A	1.2839	C41—H41	0.9300
O14—H14A	0.8200	C42—C43	1.341 (11)
O14—H14B	0.8200	C42—H42	0.9300
C1—C2	1.384 (10)	C43—C44	1.408 (11)
C1—H1	0.9300	C43—H43	0.9300
C2—C3	1.384 (11)	C44—C52	1.397 (9)
C2—H2	0.9300	C44—C45	1.434 (11)
C3—C4	1.386 (10)	C45—C46	1.324 (12)
C3—H3	0.9300	C45—H45	0.9300
C4—C12	1.398 (9)	C46—C47	1.422 (10)
C4—C5	1.448 (10)	C46—H46	0.9300
C5—C6	1.315 (11)	C47—C48	1.383 (10)
C5—H5	0.9300	C47—C51	1.412 (9)
C6—C7	1.438 (11)	C48—C49	1.389 (11)
C6—H6	0.9300	C48—H48	0.9300
C7—C8	1.389 (10)	C49—C50	1.375 (10)
C7—C11	1.410 (9)	C49—H49	0.9300
C8—C9	1.365 (11)	C50—H50	0.9300
C8—H8	0.9300	C51—C52	1.434 (9)
			
N1—Zn1—N5	97.8 (2)	C17—C18—H18	120.5
N1—Zn1—N6	97.9 (2)	C19—C18—H18	120.5
N5—Zn1—N6	77.1 (2)	C14—C19—C18	121.0 (7)
N1—Zn1—N3	163.1 (2)	C14—C19—H19	119.5
N5—Zn1—N3	96.8 (2)	C18—C19—H19	119.5
N6—Zn1—N3	93.7 (2)	N3—C21—C22	123.2 (7)
N1—Zn1—N2	77.8 (2)	N3—C21—H21	118.4
N5—Zn1—N2	168.7 (2)	C22—C21—H21	118.4
N6—Zn1—N2	93.2 (2)	C23—C22—C21	119.1 (7)
N3—Zn1—N2	89.4 (2)	C23—C22—H22	120.4
N1—Zn1—N4	93.5 (2)	C21—C22—H22	120.4
N5—Zn1—N4	95.9 (2)	C22—C23—C24	119.3 (8)
N6—Zn1—N4	167.3 (2)	C22—C23—H23	120.4
N3—Zn1—N4	76.5 (2)	C24—C23—H23	120.4
N2—Zn1—N4	94.7 (2)	C32—C24—C23	118.0 (8)
C1—N1—C12	117.1 (6)	C32—C24—C25	118.9 (8)
C1—N1—Zn1	128.1 (5)	C23—C24—C25	123.1 (8)
C12—N1—Zn1	114.8 (5)	C26—C25—C24	121.1 (8)
C10—N2—C11	118.9 (6)	C26—C25—H25	119.4
C10—N2—Zn1	129.4 (5)	C24—C25—H25	119.4
C11—N2—Zn1	111.6 (4)	C25—C26—C27	120.7 (8)
C21—N3—C32	118.3 (6)	C25—C26—H26	119.7
C21—N3—Zn1	128.2 (5)	C27—C26—H26	119.7
C32—N3—Zn1	112.5 (4)	C31—C27—C28	119.0 (8)
C30—N4—C31	118.7 (6)	C31—C27—C26	119.2 (8)
C30—N4—Zn1	128.0 (5)	C28—C27—C26	121.8 (8)
C31—N4—Zn1	112.8 (4)	C29—C28—C27	118.1 (8)
C41—N5—C52	117.9 (6)	C29—C28—H28	121.0
C41—N5—Zn1	128.6 (5)	C27—C28—H28	121.0
C52—N5—Zn1	113.1 (4)	C28—C29—C30	119.9 (8)
C50—N6—C51	118.1 (6)	C28—C29—H29	120.1
C50—N6—Zn1	128.7 (4)	C30—C29—H29	120.1
C51—N6—Zn1	113.0 (4)	N4—C30—C29	122.1 (8)
H5A—O5—H5B	105.1	N4—C30—H30	119.0
H6A—O6—H6B	103.7	C29—C30—H30	119.0
H7A—O7—H7B	103.8	N4—C31—C27	122.2 (7)
H8A—O8—H8B	128.4	N4—C31—C32	117.4 (7)
O13^i^—O9—H9B	85.2	C27—C31—C32	120.4 (7)
O13^i^—O9—H9A	149.8	N3—C32—C24	122.0 (7)
H9B—O9—H9A	123.1	N3—C32—C31	118.4 (7)
H10A—O10—H10B	118.5	C24—C32—C31	119.5 (7)
H11A—O11—H11B	116.6	O4—C33—O3	119.2 (16)
H12A—O12—H12B	105.8	O4—C33—O3'	134 (3)
O9^i^—O13—H13A	121.3	O4—C33—C34	118.4 (7)
O9^i^—O13—H13B	80.3	O3—C33—C34	122.2 (18)
H13A—O13—H13B	127.0	O3'—C33—C34	107 (2)
H9A—O14—H14A	107.9	C35—C34—C39	117.5 (8)
H9A—O14—H14B	107.7	C35—C34—C33	121.5 (7)
H14A—O14—H14B	99.6	C39—C34—C33	121.0 (8)
N1—C1—C2	123.2 (7)	C34—C35—C36	121.5 (8)
N1—C1—H1	118.4	C34—C35—H35	119.2
C2—C1—H1	118.4	C36—C35—H35	119.2
C1—C2—C3	119.1 (7)	C37—C36—C35	119.7 (8)
C1—C2—H2	120.5	C37—C36—H36	120.2
C3—C2—H2	120.5	C35—C36—H36	120.2
C2—C3—C4	119.4 (7)	C36—C37—C38	121.1 (8)
C2—C3—H3	120.3	C36—C37—Br2'	114.4 (9)
C4—C3—H3	120.3	C38—C37—Br2'	124.1 (8)
C3—C4—C12	118.7 (6)	C36—C37—Br2	122.3 (7)
C3—C4—C5	123.6 (7)	C38—C37—Br2	116.5 (7)
C12—C4—C5	117.7 (7)	C39—C38—C37	118.5 (8)
C6—C5—C4	121.7 (7)	C39—C38—H38	120.8
C6—C5—H5	119.1	C37—C38—H38	120.8
C4—C5—H5	119.1	C34—C39—C38	121.7 (8)
C5—C6—C7	122.1 (7)	C34—C39—H39	119.2
C5—C6—H6	118.9	C38—C39—H39	119.2
C7—C6—H6	118.9	N5—C41—C42	122.3 (7)
C8—C7—C11	117.7 (7)	N5—C41—H41	118.9
C8—C7—C6	123.9 (7)	C42—C41—H41	118.9
C11—C7—C6	118.3 (7)	C43—C42—C41	119.4 (7)
C9—C8—C7	119.3 (7)	C43—C42—H42	120.3
C9—C8—H8	120.3	C41—C42—H42	120.3
C7—C8—H8	120.3	C42—C43—C44	121.0 (7)
C8—C9—C10	120.0 (7)	C42—C43—H43	119.5
C8—C9—H9	120.0	C44—C43—H43	119.5
C10—C9—H9	120.0	C52—C44—C43	116.3 (7)
N2—C10—C9	122.3 (7)	C52—C44—C45	118.5 (7)
N2—C10—H10	118.8	C43—C44—C45	125.2 (7)
C9—C10—H10	118.8	C46—C45—C44	121.6 (7)
N2—C11—C7	121.7 (6)	C46—C45—H45	119.2
N2—C11—C12	119.3 (6)	C44—C45—H45	119.2
C7—C11—C12	119.1 (6)	C45—C46—C47	122.1 (7)
N1—C12—C4	122.5 (6)	C45—C46—H46	118.9
N1—C12—C11	116.6 (6)	C47—C46—H46	118.9
C4—C12—C11	121.0 (6)	C48—C47—C51	118.3 (6)
O2—C13—O1	130.0 (13)	C48—C47—C46	123.7 (7)
O2—C13—O1'	114 (2)	C51—C47—C46	118.0 (7)
O2—C13—C14	119.1 (7)	C47—C48—C49	118.4 (7)
O1—C13—C14	110.9 (12)	C47—C48—H48	120.8
O1'—C13—C14	127 (2)	C49—C48—H48	120.8
C19—C14—C15	118.5 (7)	C50—C49—C48	120.2 (8)
C19—C14—C13	121.1 (7)	C50—C49—H49	119.9
C15—C14—C13	120.4 (6)	C48—C49—H49	119.9
C14—C15—C16	121.4 (7)	N6—C50—C49	122.7 (7)
C14—C15—H15	119.3	N6—C50—H50	118.7
C16—C15—H15	119.3	C49—C50—H50	118.7
C17—C16—C15	118.6 (7)	N6—C51—C47	122.2 (6)
C17—C16—H16	120.7	N6—C51—C52	117.7 (6)
C15—C16—H16	120.7	C47—C51—C52	120.1 (6)
C16—C17—C18	121.5 (7)	N5—C52—C44	122.9 (7)
C16—C17—Br1	118.9 (6)	N5—C52—C51	117.4 (5)
C18—C17—Br1	119.6 (5)	C44—C52—C51	119.6 (6)
C17—C18—C19	118.9 (7)		

Symmetry codes: (i) −*x*+1, −*y*+2, −*z*.

### Hydrogen-bond geometry (Å, °)

**Table:**

*D*—H···*A*	*D*—H	H···*A*	*D*···*A*	*D*—H···*A*
O5—H5A···O2	0.82	1.90	2.700 (11)	164
O5—H5B···O6^ii^	0.82	2.23	2.676 (11)	114
O6—H6A···O3	0.82	1.97	2.763 (2)	162
O6—H6A···O3'	0.82	2.03	2.767 (5)	150
O6—H6B···O8^iii^	0.82	2.20	2.788 (5)	129
O7—H7A···O5^ii^	0.82	1.97	2.787 (10)	176
O7—H7B···O4	0.82	1.89	2.687 (11)	163
O8—H8A···O3	0.82	2.04	2.803 (2)	155
O8—H8A···O3'	0.82	1.99	2.789 (2)	166
O8—H8B···O1^iv^	0.82	2.19	2.862 (5)	139
O8—H8B···O1'^iv^	0.82	1.96	2.685 (6)	146
O10—H10A···O11^v^	0.82	2.24	2.806 (2)	126
O10—H10B···O2	0.82	2.11	2.739 (2)	134
O11—H11A···O5	0.82	2.27	2.826 (5)	126
O11—H11B···O10^v^	0.82	2.33	2.806 (2)	117
O12—H12A···O13^ii^	0.82	2.50	2.981 (5)	118
O12—H12B···O4	0.82	2.29	2.786 (2)	119
O13—H13A···O12^vi^	0.82	2.19	2.796 (5)	131
O13—H13B···O7^ii^	0.82	1.95	2.746 (2)	165

Symmetry codes:  (ii) −*x*+2, −*y*+3, −*z*+1; (iii) −*x*+1, −*y*+2, −*z*+1; (iv) *x*−1, *y*−1, *z*; (v) −*x*+2, −*y*+3, −*z*; (vi) *x*, *y*+1, *z*−1.
